# A National Survey Comparing Patients’ and Transplant Professionals’ Research Priorities in the Swiss Transplant Cohort Study

**DOI:** 10.3389/ti.2022.10255

**Published:** 2022-05-18

**Authors:** Sonja Beckmann, Oliver Mauthner, Liz Schick, Jessica Rochat, Christian Lovis, Annette Boehler, Isabelle Binet, Uyen Huynh-Do, Sabina De Geest, the Psychosocial Interest Group, Sabina De Geest, the Swiss Transplant Cohort Study, Patrizia Amico

**Affiliations:** ^1^ Department Public Health, Institute of Nursing Science, University of Basel, Basel, Switzerland; ^2^ Center Clinical Nursing Science, University Hospital Zurich, Zurich, Switzerland; ^3^ University Department of Geriatric Medicine Felix Platter, Basel, Switzerland; ^4^ Swisstransplant, Bern, Switzerland; ^5^ Faculty of Medicine, University of Geneva, Geneva, Switzerland; ^6^ Division of Medical Information Sciences, University Hospitals of Geneva, Geneva, Switzerland; ^7^ University of Zurich, Zürich, Switzerland; ^8^ Service of Nephrology and Transplantation Medicine, Cantonal Hospital, St. Gallen, Switzerland; ^9^ Department of Nephrology and Hypertension, Inselspital, University of Bern, Bern, Switzerland; ^10^ Academic Center for Nursing and Midwifery, Department of Public Health and Primary Care, KU Leuven, Leuven, Belgium

**Keywords:** organ transplantation, patient involvement, research priorities, qualitative methods, registry-based study

## Abstract

We aimed to identify, assess, compare and map research priorities of patients and professionals in the Swiss Transplant Cohort Study. The project followed 3 steps. 1) Focus group interviews identified patients’ (*n* = 22) research priorities. 2) A nationwide survey assessed and compared the priorities in 292 patients and 175 professionals. 3) Priorities were mapped to the 4 levels of Bronfenbrenner’s ecological framework. The 13 research priorities (financial pressure, medication taking, continuity of care, emotional well-being, return to work, trustful relationships, person-centredness, organization of care, exercise and physical fitness, graft functioning, pregnancy, peer contact and public knowledge of transplantation), addressed all framework levels: patient (*n* = 7), micro (*n* = 3), meso (*n* = 2), and macro (*n* = 1). Comparing each group’s top 10 priorities revealed that continuity of care received highest importance rating from both (92.2% patients, 92.5% professionals), with 3 more agreements between the groups. Otherwise, perspectives were more diverse than congruent: Patients emphasized patient level priorities (emotional well-being, graft functioning, return to work), professionals those on the meso level (continuity of care, organization of care). Patients’ research priorities highlighted a need to expand research to the micro, meso and macro level. Discrepancies should be recognized to avoid understudying topics that are more important to professionals than to patients.

## Graphical Abstract

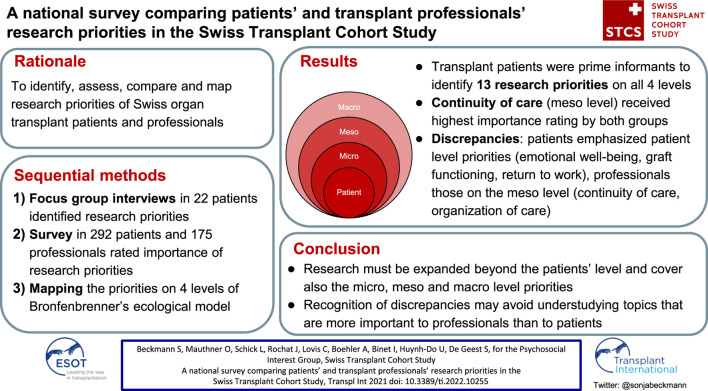

## Introduction

Setting research priorities with patient involvement is key to optimizing resources, reducing research waste and producing relevant and warranted evidence that improves not only clinical practice but also the quality of life of those affected. When setting research priorities, an increasing number of initiatives promote the involvement not only of clinicians and researchers but also of patients and other stakeholders (1–7). These efforts have been essential in determining the research agenda (1), conducting research toward the needs of those who live with a certain condition (8), performing research with the greatest public health benefit and enhancing the societal return-on-investment of research funding (9, 10). Within the research team, patients contribute perspectives that may be based on the lived experience and therefore complement the scientific view. former Chief Medical Officer for England, Professor Dame Sally Davis, aptly highlighted the beneficial effect of diverse perspectives: “No matter how complicated the research, or how brilliant the researcher, patients and the public always offer unique, invaluable insights. Their advice when designing, implementing and evaluating research invariably makes studies more effective, more credible and often more cost efficient as well” (11).

In the transplant setting, a large international study revealed that patients and clinicians differ considerably in their opinions about relevant research outcomes that should be assessed (12). The discrepancy highlights the necessity to thoroughly understand patients’ needs and opinions in order to add their perspective in the process of setting research priorities. A systematic review examined 28 transplant research priority setting projects involving different stakeholders such as patients, healthcare providers, policymakers and researchers (13). Tong et al. found that only nine projects (32%) reported patient or caregiver involvement, restricted to projects in kidney and heart transplantation. The nine projects used different methodologies to identify research priorities such as surveys, interviews or workshop discussions. Importantly, only one project started the priority setting process from the patients’ perspective (14).

The Swiss Transplant Cohort Study (STCS), a nation-wide prospective cohort study started in 2008, has currently involved more than 6300 patients. The STCS collects a broad set of biomedical, genetic and psychosocial variables, including patient-reported outcomes, before and after transplantation (15, 16). In 2017, driven by the international and national call for patient involvement in research priority setting (1, 4, 8), the STCS launched a patient involvement project. This study is part of that project, which followed the stages of the research cycle as recommended by the INVOLVE report and started to first identify and prioritize research topics (1). Given that an individuum is not isolated but surrounded by a wider community and society, Bronfenbrenner ecological framework suggests four levels (i.e., patient, micro, meso and macro level) to examine interactions and relationships (17, 18). Therefore, the aims of this study were to identify, assess, and compare the research priorities of Swiss transplant patients and transplant professionals and to map the priorities according to Bronfenbrenner’s framework.

## Patients and Methods

### Design

This study was a sequential multi-methods project. First, we conducted focus group interviews with organ transplant patients to identify research priorities from the patient perspective. Second, we conducted a survey to assess and compare the importance of research priorities in transplant patients and transplant professionals. Third, we conceptually mapped the research priorities according to the ecological framework by Bronfenbrenner (17, 18). The study received a declaration of no objection from Swissethics (EKNZ Req-2017-00279).

### Part 1: Focus Group Interviews With Patients

#### Sample and Setting

We conducted 3 focus group interviews to identify research priorities. To facilitate the journey to the interviews, recipients could choose from three locations (Zurich, Basel or Geneva). Inclusion criteria were age ≥18 years and having received a multi-organ transplant or a single kidney, liver, heart or lung transplantation. People who were not able to speak German or French were excluded.

#### Data Collection and Analysis

The interviews were conducted in April and May 2017. Eligible participants of all transplant centers were asked by the local STCS data manager or members of the study team to participate. In advance, they received from the researchers oral and written information about the purpose of the discussion, the voluntary nature of their participation and the use of their contributions. Prior to the discussion, all participants were informed, that the discussion content would be treated confidentially and were asked to agree to an audio-recording of the interview.

At the beginning of each interview, participants were encouraged to talk to each other, and interactions within the group were stimulated. The discussions were guided by a semi-structured guideline, which was sent to the participants in advance to facilitate preparation. The guideline included three open-ended questions: 1) What is important for you, or what concerns do you have when dealing with your transplantation? 2) Which questions should researchers focus on to improve life with a transplantation? 3) Which topics are important for you following your transplantation? Probe questions on specific transplant topics (e.g., psychosocial issues, psychological and social support, comorbidities) guided further discussions if necessary.

The knowledge mapping technique was used for analysis, allowing an organized, condensed and visualized presentation of the issues emerging from each focus group interview (19, 20). While the main moderator guided the interview, the co-moderator identified and grouped important topics in the maps. At the end of each interview, the co-moderator explained and summarized the knowledge maps to the participants. The visualization highlighted relationships and allowed related themes to be developed. This procedure and the resulting discussion served to validate the topics and was considered as the first step of data analysis. Afterwards, the knowledge maps of all three focus group interviews were reviewed and analyzed by research team members to identify common topics and research priorities.

### Part 2: Survey Among Patients and Professionals

The focus groups generated 13 research priorities, represented by 34 example statements. The 34 statements formed one section of a 95-item questionnaire on research priorities and patient involvement in transplant research. The questionnaire’s two other sections covered the importance of patient involvement (5 items) and factors to be assessed in STCS (56 items), which were not the focus of this analysis. The questionnaire was translated by native speakers from German to English and French.

#### Setting and Sample

The questionnaire was distributed among a convenience sample of patients and professionals in all six transplant centers and their respective solid organ transplant outpatient clinics in Switzerland. Inclusion criteria for the patients were: age ≥18 years, having received a multi-organ transplant or a single kidney, liver, heart or lung transplantation. Patients in the immediate perioperative period, meaning those, who were still hospitalized after transplantation, were excluded. Inclusion criteria for the professionals were: age ≥18 years, being a member of the STCS (i.e., researcher, data manager) or being a professional who cares for transplant patients in one of the six transplant centers (i.e., nurse, physician). Patients and professionals unable to speak German, English or French were excluded.

#### Data Collection and Management

Data were collected in November and December 2017. Patients were recruited and informed about the study during their follow-up appointment in the transplant centers’ outpatient clinics by the nurses and physicians. Interested patients received a hard copy of the questionnaire in their preferred language and a pre-stamped envelope to return the questionnaire to the study team. Patients who preferred to participate online received a link to the electronic version of the survey.

All professionals in the STCS and in the six transplant centers were invited via e-mail to participate in the online survey in their preferred language. The e-mail with written study information and the link was distributed by the key stakeholders in the STCS and each transplant center. At the end of the data collection, the online data were transferred to a statistical software program. Two team members individually entered the data of the paper questionnaires in the statistical software program and double checked each entry for potential mistakes.

#### Variables and Measurements

The 34 items in our survey were rated regarding their importance for transplant research on a 9-point Likert-scale from 1 (not at all important) to 9 (very important) with the additional answer option “unsure”. The ratings from the continuous scale were dichotomized (cutoff at 7) for further analysis: items with values ≥ 7 were considered “important” to the participants. For each item, we noted the proportion of the “important” rating. The answer option “unsure” was considered as a missing value. The following general information was collected from patients: gender, age in years, transplanted organ, date of first transplant and transplant center; and from professionals: gender, age in years, years working in the field of transplantation, profession and specialization.

#### Data Analysis

Descriptive statistics included frequencies and percentages, mean and standard deviation, as well as median and interquartile range (IQR) as appropriate. Seventeen items had missing values > 10%, which were not imputed. Discrepancies among patients and professionals in importance scores were calculated by subtraction. Scores were compared using a Chi square test. A two-tailed *p*-value <0.05 was considered statistically significant. Statistical analyses were conducted using IBM SPSS Version 25.0 for Mac (Armonk, NY: IBM Corp).

### Part 3: Mapping Transplant Research Priorities

The research priorities (and corresponding example statements) were subsequently mapped according to the 4 levels of Bronfenbrenner’s ecological framework (17, 18): Patient level was defined as individual issues and characteristics such as knowledge, attitudes or behavior. Micro level was related to social support, interpersonal relationships and interactions between patient, family and healthcare providers. Meso level represented practice patterns and characteristics of the transplant center or the health care organization where patients were treated. Macro level covered issues related to the healthcare system and at policy level.

## Results

### Part 1: Identification of Patients’ Research Priorities

Twenty-two patients participated in the focus groups (Zurich n = 7, Basel n = 10, Geneva n = 5). They had received an organ transplant between 1998 and 2017 (kidney n = 9, 43%, liver n = 6, 29%, heart n = 4.19% and lung n = 2, 9%). The majority was female (n = 12, 57%) and the mean age was 53 years. Patients discussed a broad variety of issues, with congruous issues being discussed in each of the 3 focus groups. We identified 13 research priorities, represented by 34 example statements: financial pressure (n = 5 example statements); medication taking, continuity of care (each n = 4); emotional well-being, return to work, trustful relationships, person-centeredness, organization of care (each n = 3); exercise and physical fitness (n = 2); graft functioning, pregnancy, peer contact, and public knowledge of transplantation (each n = 1). A list of all research priorities and example statements is provided in the supplemental digital content (Supplementary Table S1).

### Part 2: Assessment and Comparison of Patient and Professional Research Priorities

Across the 6 transplant centers, 16 outpatient clinics recruited patients. One kidney transplant outpatient clinic did not participate due to high workload. Of the 735 questionnaires distributed to patients, 292 were returned (response rate 39.7%). The online survey was completed by 175 professionals. The response rate was not calculated given the unknown denominator. Patient and professional characteristics are shown in Table 1.

**TABLE 1:** Patient and professional characteristics.

	Valid n	Patients, n = 292
Male gender, n (%)	256	171 (58.6)
Age in years, median (IQR)	246	58 (27-65)
Time after Tx in years, median (IQR)	263	4.37 (0.6-12.4)
Tx organ	255	
Kidney, n (%)		160 (55)
Liver, n (%)		48 (16)
Heart, n (%)		38 (13)
Lung, n (%)		1 (0.3)
Other, n (%)		5 (2)
Combined, n (%)		3 (1)
Tx center	254	
Basel, n (%)		79 (27)
Zurich, n (%)		48 (16)
Bern, n (%)		44 (15)
Lausanne, n (%)		37 (13)
St. Gallen, n (%)		22 (8)
Geneva, n (%)		24 (8)
	**Valid n**	**Professionals, n = 175**
Male gender, n (%)	158	81 (46)
Age in years, median (IQR)	158	42.5 (36-51)
Time working in Tx in years, median (IQR)	158	10 (3.75-17)
Profession	158	
Physician, n (%)		119 (68)
Nurse, n (%)		17 (10)
Researcher, n (%)		10 (6)
Data manager, n (%)		5 (3)
Other, n (%)		7 (4)
Specialization	157	
Nephrology, n (%)		51 (29)
Transplant surgery, n (%)		28 (16)
Hepatology, n (%)		12 (7)
Infectiology, n (%)		10 (6)
Pulmonology, n (%)		6 (3)
Cardiology, n (%)		2 (1)
Other, n (%)		48 (27)

SD: standard deviation, IQR: interquartile range, Tx: transplant.

The complete ranking of research priorities and example statements by patients and professionals is provided in the supplemental digital content (Supplementary Table S1). Table 2, with the top 10 research priorities for both groups, shows that both groups agreed in their highest rating on continuity of care (“Care begins even before the transplant takes place”), which was important to 92.2% of the patients (n = 282) and 92.5% of the professionals (n = 181). Otherwise, patients and professionals had only 3 more matches in their top 10 ratings. The overall priorities of both groups differed as patients mostly chose statements relating to emotional well-being (n = 3 statements) while professionals emphasized statements relating to continuity of care (n = 4 statements).

**TABLE 2:** The top 10 research priorities with corresponding example statements and the level of the ecological framework for each group.

Top 10 Ratings by patients (n = 292)		% (Valid n)	Top 10 Ratings by professionals (n = 175)		% (Valid n)
**1**	**Continuity of care—Meso level**	**92.2 (282)**	**1**	**Continuity of care—Meso level**	**92.5 (161)**
	Care begins even before the transplant takes place.			Care begins even before the transplant takes place.	
**2**	**Continuity of care—Meso level**	**91.2 (285)**	**2**	**Organization of care—Meso level**	**90.6 (160)**
	It’s nice if you can always call the same people at the hospital. Then they know you.			I would like a telephone number where I can get a sensible answer if I call. A point of contact where I can clarify whether I need to go to hospital or not.	
**3**	**Person-centeredness—Micro level**	**87.9 (173)**	3	**Organization of care—Meso level**	89.9 (159)
	My life does not consist solely of the transplant. A good doctor is one who sees the person as a whole, who sees you as a complete person and not just as a “transplanted organ".			I discovered that I did not have a contact person at the hospital. There is nobody that I can relate to, and I miss that.	
4	**Public knowledge of transplantation—Macro level**	82 (272)	**4**	**Continuity of care—Meso level**	**85.7 (161)**
	The general public needs to be better educated about organ transplantation. People have strange ideas.			It’s nice if you can always call the same people at the hospital. Then they know you.	
5	**Emotional well-being—Patient level**	81.4 (269)	**5**	**Person-centeredness—Micro level**	**83.8 (160)**
	How you deal with the illness is important. How you find a balance between anxiety, the consequences of the transplant and the desire to live.			My life does not consist solely of the transplant. A good doctor is one who sees the person as a whole, who sees you as a complete person and not just as a “transplanted organ".	
6	**Graft functioning—Patient level**	78.3 (263)	6	**Continuity of care—Meso level**	83.4 (157)
	I worry about how long my graft will last. I don’t know what to expect. I’d like to see research focused on ways to make grafts last longer.			I had a new doctor every time. He had never seen me before and I had to explain everything all over again. This usually took up most of the appointment time.	
7	**Emotional well-being—Patient level**	77.2 (272)	7	**Trustful relationships—Micro level**	82.5 (160)
	It is my motivation: what progress can I see for myself from day to day. It just needs a lot of discipline. Otherwise, it doesn’t work.			In hospital they said I should go to my GP. But he is so overwhelmed with my case that it makes me even more uncertain, and I have lost confidence in the hospital and in my GP.	
8	**Emotional well-being—Patient level**	76.9 (268)	8	**Continuity of care—Meso level**	81.1 (159)
	Not everybody, especially younger people, can master it in the same way. Attention should be paid to psychological care as well as to medical care.			Prior to the transplant there is too little information about what happens afterwards.	
9	**Return to work—Patient level**	73.8 (244)	9	**Return to work—Patient level**	79.9 (159)
	Many young people who have not worked or were unable to do training prior to the transplant later have great difficulty getting back into work.			I am still very tired during the day and I have difficulty concentrating. Now I’ve been given notice and the application for disability insurance is pending. But at 56 you’re really gone - and I don’t know what will happen now.	
**10**	**Organization of care—Meso level**	**73.6 (269)**	10	**Exercise and physical fitness—Patient level**	76.9 (160)
	I would like a telephone number where I can get a sensible answer if I call. A point of contact where I can clarify whether I need to go to hospital or not.			Since the transplant, exercise is very important to me. I enjoy it immensely.	

The 4 matching example statements among the groups are highlighted with bold rank numbers and % values. The shades of gray represent the ecological framework levels.

**FIGURE 1:**
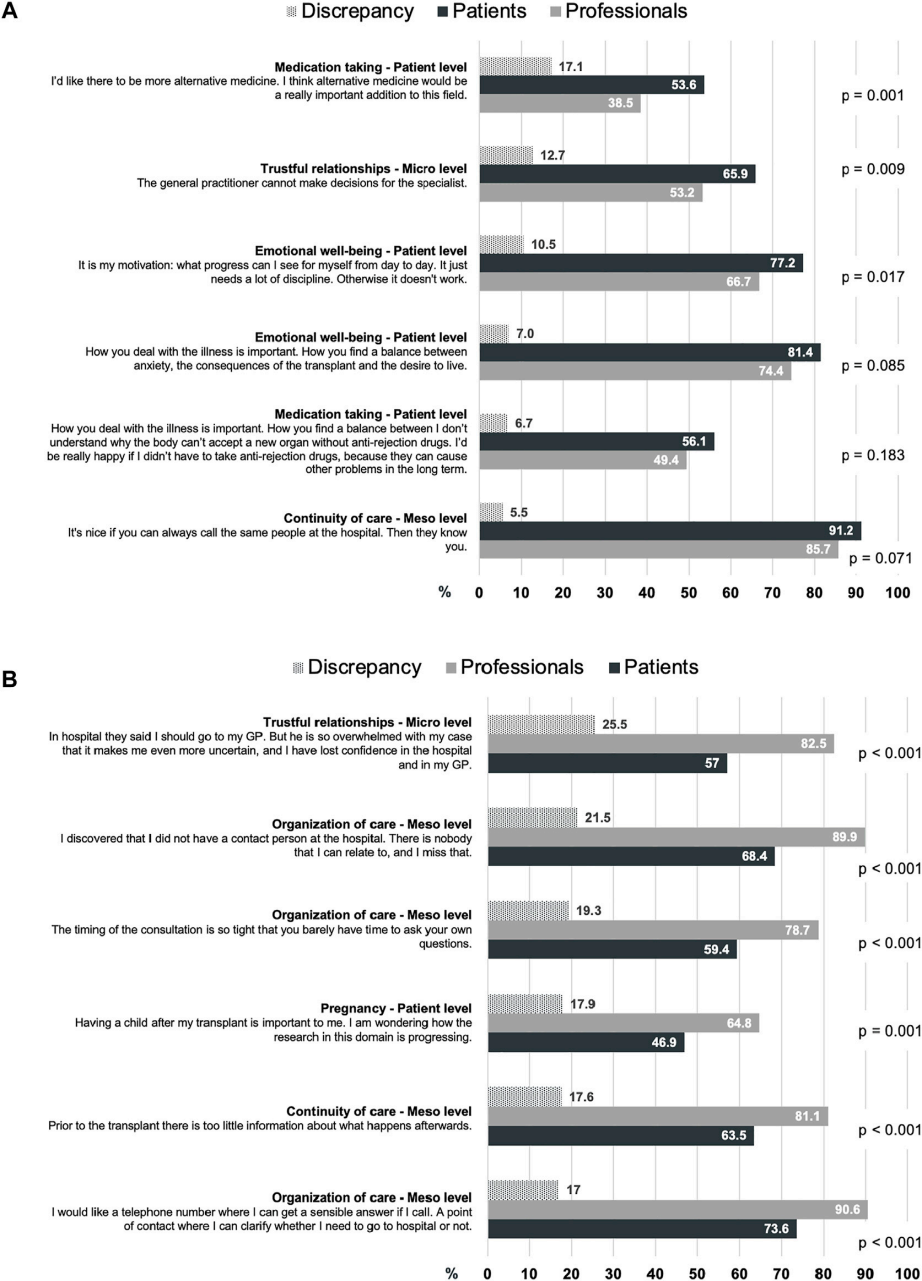
The 6 highest discrepancies in the rating on research priorities and statements from **(A)** the patient perspective and **(B)** the professional perspective, in descending order.

Figure 1 shows the 6 highest ranked discrepancies from each perspective. From the patient perspective, the highest discrepancy in research priorities (17.1%) was medication taking, which was important to 53.6% of the patients and to only 38.5% of the professionals (Figure 1A). From the professional perspective, the highest discrepancy in research priorities (25.5%) was trustful relationships, which was important to 82.5% of the professionals and to only 57% of the patients (Figure 1B).

### Part 3: Mapping Research Priorities According to the Ecological Framework

**FIGURE 2:**
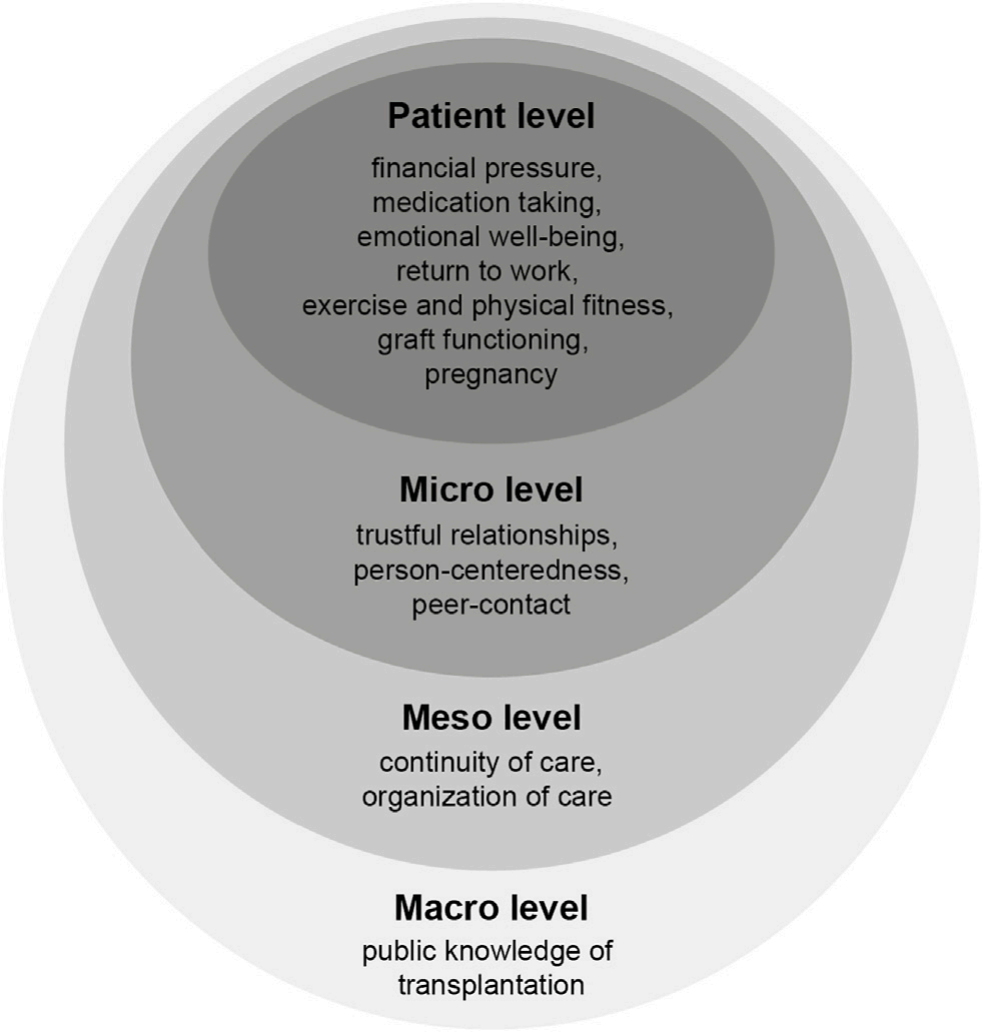
The 13 research priorities assigned to the 4 levels of the ecological framework.

Figure 2 shows the mapping of the 13 research priorities addressing all 4 levels of the ecological framework: 7 patient level priorities (financial pressure, medication taking, emotional well-being, return to work, exercise and physical fitness, graft functioning, pregnancy), 3 micro level priorities (trustful relationships, person-centeredness, peer contact), 2 meso level priorities (continuity of care, organization of care), and 1 macro level priority (public knowledge of transplantation).

Patients and professionals focused on different research priority levels (Table 2). Within the top 10 research priorities for each group, the biggest proportion of patients’ priorities was on the patient level (n = 5), such as emotional well-being, graft functioning and return to work. In addition, patients’ priorities covered all 4 levels of the ecological model. Professionals’ priorities were most often on the *meso level* (n = 6), such as continuity of care and organization of care, while they only chose 2 patient level priorities such as return to work and exercise and physical fitness.

The discrepancies between the groups revealed the same distribution of research priority levels (Figure 1). From the patient perspective, 4 out of the 6 discrepancies were related to the patient level priorities emotional well-being and medication taking (Figure 1A). From the professional perspective, 4 out of the 6 discrepancies were related to the meso level priorities organization of care and continuity of care (Figure 1B).

## Discussion

Our project identified 13 transplant research priorities covering a broad range of topics on all levels of the ecological framework. Setting research priorities informed by the patients’ perspectives has gained increased importance over the last decade, also in transplantation (13). Our findings strengthen and expand this movement, especially as we focused on transplant patients as prime informants to determine research priorities.

We chose this approach to maximize the patients’ inputs from the beginning; however, there are other methods. The James Lind Alliance, for example, suggested identifying research priorities based on the equal voices of various stakeholders (8). So far, the approach of working with a mixed stakeholder group instead of patients only seemed to be the more common practice in research into priorities for solid organ transplantation. A systematic review has examined 28 research priority setting projects, 27 of which identified priorities based on the combined inputs from patients, caregivers, clinicians, researchers or policy makers (13). While the inclusion of diverse stakeholders is commendable, the authors also observed in the included studies a lack of details on the process of identifying the research priorities. Using a reporting checklist such as the GRIPP2 (Guidance for Reporting Involvement of Patients and the Public) (21) may enhance the quality of reporting and provide transparent information on the process of stakeholder involvement.

We mapped the research priorities according to the ecological model to enhance the interpretation of our results. Overall, the majority of our survey’s example statements and research priorities were assigned to the patient level. The importance of patient-oriented topics was also highlighted by other projects, which primarily identified patient level priorities such as transplant outcomes, graft or recipient complications, immunosuppressive medication, fertility/pregnancy or organ donation criteria (13, 22). However, our results add to previous evidence because patients and professionals highlighted the need to expand the research to the micro, meso and macro level. Few transplantation studies integrated the transplant center or healthcare system level perspectives to examine transplant outcomes. A recent study used data from a multi-continental project in heart transplantation to examine nonadherence with immunosuppressive medication (23). Besides patient level factors, the authors also considered variables on the micro level (e.g., social support, trust in the healthcare team), the meso level (e.g., duration of visit in the outpatient clinic, care by a multidisciplinary team) and the macro level (e.g., health insurance covering costs for immunosuppressants). The multiple logistic regression identified 6 correlates from all ecological levels as associated with immunosuppressant nonadherence, which broadened the picture and increased understanding of medication nonadherence. We therefore encourage future transplant studies to follow this inclusive approach. Considering the micro, meso and macro level perspectives is likely to enlarge the evidence and therefore potentially improve patient outcomes and quality of care.

Another finding from our study supports the expansion beyond patient level factors because both parties agreed on their most important research priority continuity of care, which belongs to the meso level. Continuity of care is a broad concept, which can be characterized by three elements: longitudinal care with as few professionals as possible, a caring patient-professional relationship and coordinated care (24). It relates to the other meso level priority, organization of care, which was second most chosen by professionals. Patients and professionals therefore identified the need to consider the principles of chronic illness management in transplant research. In numerous chronically ill populations, the re-organization of care delivery according to the components of chronic illness management has improved outcomes such as reducing hospital admissions, improving health behaviors or a better quality of life (25). As researchers and clinicians have already called to adapt follow-up care to the principles of chronic illness management, which better reflects the complex needs of solid organ transplant recipients (26), our results emphasize the importance of accompanying this process in transplantation by research.

Our results on the ranking of research priorities, however, revealed perceptions to be more diverse than congruent among patients and professionals. Within the top 10, the groups shared only 4 common research priorities, and our immediate comparison of research priorities of each groups’ perspectives revealed additional discrepancies. Overall, patients chose more patient level priorities, professionals those on the meso level. Patient level priorities in our study covered various elements such as graft functioning, emotional well-being and return to work, thereby highlighting the need to expand transplant research beyond purely clinical or medical topics to psychosocial topics. Prioritizing psychosocial topics was also a finding in a systematic review, although this research priority scored comparatively low in their ranking since only 7 of the 28 reviewed studies mentioned psychosocial and lifestyle topics (13).

Dissenting views on research priorities among stakeholders have also been observed in other priority setting projects. Knight et al. used the James Lind Alliance method to identify and prioritize unanswered research questions in the field of kidney transplantation (22). Professionals and non-professionals initially identified 497 questions covering all parts of transplantation. After a process of surveying, grouping, refining and validating, a final set of 25 top ranked questions was discussed in a workshop with patients, carers and healthcare professionals. The groups agreed on the importance of improving long-term transplant outcomes; however, patients prioritized questions about immunosuppression, organ preservation and equity of access while professionals emphasized medical aspects such as the assessment of patient and organ suitability as well as the management of antibody mediated rejection. A systematic review reported the same pattern with patients focusing on person-centered topics (e.g., patient and family education, reducing side-effects of medication, quality of life) and professionals prioritizing technical or policy aspects of transplantation (e.g., HLA antibodies and sensitization, allocation, pharmacokinetics of immunosuppression) (13). While our study also revealed discrepant views among patients and professionals, the topics differed from the previous examples. The reason might be that, in our project, the research priorities were initially determined by patients. Since the survey did, therefore, not include procedures, medical or technical topics related to transplantation, participants, and especially professionals, could not choose research priorities from these domains.

Regardless of whether the process of identifying research priorities was initiated by mixed stakeholders or patients only, an important finding from our study and previous projects is that discrepancies between stakeholders occur and should therefore be recognized and used to an advantage. Emphasizing research priorities with high importance to patients but less importance to professionals may reduce the risk of understudying those issues in research. This emphasis also strengthens the necessity to involve patients early in the research process to combine the perspectives of lived experience and science (1). Indeed, combining complementary views in research priority settings can be positive and productive as it reduces the risk of a mismatch between the research being conducted and the research expected by all parties (1, 8). However, it seems as if this combining is easier said than done. A recent study found that only 27% of the published articles in two main transplant journals considered research priorities as identified by patients, caregivers and researchers (27). More effort will be needed if the priorities and the research conducted are to be better matched. Importantly, the transplant community already started activities to support this movement. The newly established European Transplant Patient Organization, initiated by the European Society for Organ Transplantation, is considered to function as a platform to support mutual understanding, learning and collaborative partnership between transplant professionals and solid organ recipients (28).

Our study was conducted within the research framework of the STCS, and the results will shape the future STCS research agenda towards more diverse perspectives. We identified research priorities on each level of the ecological model. They will now support the development of specific research questions, guided by specific evidence and the needs of each solid organ transplant group separately. This process will again involve transplant patients because evidence suggests that patient involvement enhances the significance of research projects and the impact of study findings (29–31).

While the STCS will take further actions based on the results of this study, some limitations should be noted. First, dichotomizing the answer categories might have resulted in a loss of variability compared to using mean values. Second, perspectives from participants speaking languages other than German, French or English are missing. Especially patients from other cultural or ethnic backgrounds probably deal with different issues, which were not highlighted in our nationwide survey. This perspective should be examined in future research.

In conclusion, patients identified research priorities, which were compared and assessed in a nationwide survey with patients and professionals and mapped according to the ecological framework. Our results highlight the need to expand research to cover not only patient level but also micro, meso and macro level topics. However, comparing the research priorities revealed diverse perspectives that should be acknowledged. Patients focused on patient level priorities related to psychosocial issues while professionals emphasized meso level priorities related to the principles of chronic illness management. Our findings add a crucial patient perspective to the STCS research agenda and the broader transplant research community. Combining the perspectives of lived experience and science will facilitate future research that is of high priority to both patients and professionals.

## The Members of the Psychosocial Interest Group

Sabina De Geest, Kris Nadine Beerli, Sabina De Geest, Kris Denhaerynck, Petra Künzler, Lynn Leppla, Janette Ribaut (University of Basel); Annette Boehler, Michael Koller (University Hospital Basel); Oliver Mauthner (University of Basel, University Department of Geriatric Medicine Felix Platter); Lut Berben (University Children’s Hospital Basel); Uyen Huynh-Do (University Hospital Inselspital Bern); Aurelia Mercay (University Hospital Geneva); Karine Hadaya (University Hospital Geneva); Annina Seiler, Joëlle Lynn Dreifuss (University Hospital Zurich); Isabelle Binet (Cantonal Hospital St. Gallen); Patrizia Künzler-Heule (University of Basel, Cantonal Hospital St.Gallen); Hanna Burkhalter (Kantonsspital Graubünden), Marian Struker (Kinderspital Zürich), Sonja Beckmann (University of Basel, University Hospital Zurich), Christian Rothlisberger (patient representative)

## The Members of the Swiss Transplant Cohort Study

Patrizia Amico, Andres Axel, John-David Aubert, Vanessa Banz, Beckmann Sonja, Guido Beldi, Christoph Berger, Ekaterine Berishvili, Isabelle Binet, Pierre-Yves Bochud, Sanda Branca, Heiner Bucher, Thierry Carrel, Emmanuelle Catana, Yves Chalandon, Sabina De Geest, Olivier de Rougemont, Michael Dickenmann, Joëlle Lynn Dreifuss, Michel Duchosal, Thomas Fehr, Sylvie Ferrari-Lacraz, Nicola Franscini, Christian Garzoni, Paola Gasche Soccal, Christophe Gaudet, Emiliano Giostra, Déla Golshayan, Nicolas Goossens, Karine Hadaya, Jörg Halter, Dominik Heim, Christoph Hess, Sven Hillinger, Hans Hirsch, Patricia Hirt, Günther Hofbauer, Uyen Huynh-Do, Franz Immer, Michael Koller (Head of the data center), Mirjam Laager, Bettina Laesser, Roger Lehmann, Alexander Leichtle, Christian Lovis, Oriol Manuel, Hans-Peter Marti, Pierre Yves Martin, Michele Martinelli, Valérie McLin, Katell Mellac, Aurélia Merçay, Karin Mettler, Nicolas Mueller (Chairman Scientific Committee), Antonia Müller, Thomas Müller, Ulrike Müller-Arndt, Beat Müllhaupt, Mirjam Nägeli, Graziano Oldani, Manuel Pascual (Executive office), Klara Posfay-Barbe, Juliane Rick, Anne Rosselet, Simona Rossi, Silvia Rothlin, Frank Ruschitzka, Urs Schanz, Stefan Schaub, Aurelia Schnyder, Macé Schuurmans, Thierry Sengstag, Federico Simonetta, Susanne Stampf, Jürg Steiger (Head, Excecutive office), Guido Stirniman, Ueli Stürzinger, Christian Van Delden (Executive office), Jean-Pierre Venetz, Jean Villard, Madeleine Wick (STCS coordinator), Markus Wilhlem, Patrick Yerly

## Data Availability

The raw data supporting the conclusion of this article will be made available by the authors, without undue reservation.

## Abbreviations

STCS, Swiss Transplant Cohort Study.
