# Supplementary material for: A National Survey Comparing Patients’ and Transplant Professionals’ Research Priorities in the Swiss Transplant Cohort Study
Source: Transpl Int. 2022 May 18;35:10255. doi: 10.3389/ti.2022.10255 (PMC9156624; doi:10.3389/ti.2022.10255)
Supplement: Supplementary file 1 [file Table1.DOCX]

**Supplement Table 1.** **The ranking of all 13 transplant research priorities and 34 example statements.**

The rank numbers show the importance rating from the perspective of each group, patients and professionals. The difference between the ratings was calculated by subtracting the professionals’ ratings from the patients’ ratings. The importance rating percentages marked in bold are the 10 highest ranked statements by each group. Significant differences between patients and professionals are highlighted in bold.

| **Research priorities** | **Example statements** | **Level of the eco-logical frame-work** | **Rank No.** | **Importance rating**  **by patients,**  **n = 292** | | **Rank No.** | **Importance rating**  **by professionals,**  **n = 175** | | **Difference between ratings** | **P value** |
| --- | --- | --- | --- | --- | --- | --- | --- | --- | --- | --- |
|  |  |  |  | Valid n | % |  | Valid n | % | % |  |
| Continuity of care | Care begins even before the transplant takes place. | Meso | **1** | 282 | **92.2** | **1** | 161 | **92.5** | -0.3 | 0.895 |
| Continuity of care | It's nice if you can always call the same people at the hospital. Then they know you. | Meso | **2** | 285 | **91.2** | **4** | 161 | **85.7** | 5.5 | 0.071 |
| Person- centeredness | My life does not consist solely of the transplant. A good doctor is one who sees the person as a whole, who sees you as a complete person and not just as a "transplanted organ". | Micro | **3** | 173 | **87.9** | **5** | 160 | **83.8** | 4.1 | 0.282 |
| Public knowledge of transplantation | The general public needs to be better educated about organ transplantation. People have strange ideas. | Macro | **4** | 272 | **82.0** | 13 | 160 | 77.5 | 4.5 | 0.257 |
| Emotional well-being | How you deal with the illness is important. How you find a balance between anxiety, the consequences of the transplant and the desire to live. | Patient | **5** | 269 | **81.4** | 21 | 160 | 74.4 | -9.2 | 0.085 |
| Graft functioning | I worry about how long my graft will last. I don’t know what to expect. I’d like to see research focused on ways to make grafts last longer. | Patient | **6** | 263 | **78.3** | 16 | 157 | 75.8 | 2.5 | 0.549 |
| Emotional well-being | It is my motivation: what progress can I see for myself from day to day. It just needs a lot of discipline. Otherwise, it doesn't work. | Patient | **7** | 272 | **77.2** | 29 | 159 | 66.7 | 10.5 | **0.017** |
| Emotional well-being | Not everybody, especially younger people, can master it in the same way. Attention should be paid to psychological care as well as to medical care. | Patient | **8** | 268 | **76.9** | 14 | 159 | 77.4 | -0.5 | 0.907 |
| Return to work | Many young people who have not worked or were unable to do training prior to the transplant later have great difficulty getting back into work. | Patient | **9** | 244 | **73.8** | 11 | 160 | 78.8 | -5.0 | 0.253 |
| Organization of care | I would like a telephone number where I can get a sensible answer if I call. A point of contact where I can clarify whether I need to go to hospital or not. | Meso | **10** | 269 | **73.6** | **2** | 160 | **90.6** | -17.0 | **<0.001** |
| Return to work | I am still very tired during the day and I have difficulty concentrating. Now I've been given notice and the application for disability insurance is pending. But at 56 you're really gone - and I don't know what will happen now. | Patient | 11 | 255 | 71.8 | **10** | 159 | **79.9** | -8.1 | 0.064 |
| Continuity of care | I had a new doctor every time. He had never seen me before and I had to explain everything all over again. This usually took up most of the appointment time. | Meso | 12 | 276 | 71.7 | **6** | 157 | **83.4** | -11.7 | **0.006** |
| Medication taking | I would be extremely grateful if medicines could be found that you don't have to take so frequently. | Patient | 13 | 272 | 71.3 | 24 | 158 | 72.8 | -1.5 | 0.745 |
| Exercise and physical fitness | After the transplant I had practically no muscles left. It took a year for me to become properly fit again. | Patient | 14 | 265 | 71.3 | 18 | 160 | 75.6 | -4.3 | 0.333 |
| Person- centeredness | I would like the doctors to show much more empathy and more understanding for what a transplant really means for patients. | Micro | 15 | 274 | 70.4 | 19 | 160 | 75.0 | -4.6 | 0.307 |
| Return to work | All you get is rejections. At the interviews it was often a question of: "You've had a transplant and you're probably often ill, we don't want that." | Patient | 16 | 243 | 69.1 | 15 | 159 | 76.1 | -7.0 | 0.129 |
| Financial pressure | It cannot be taken for granted that you can afford to be ill. | Patient | 17 | 257 | 68.9 | 30 | 158 | 65.2 | 3.7 | 0.437 |
| Organization of care | I discovered that I did not have a contact person at the hospital. There is nobody that I can relate to, and I miss that. | Meso | 18 | 275 | 68.4 | **3** | 159 | **89.9** | -21.5 | **<0.001** |
| Financial pressure | Until the invalidity insurance has decided what it's doing, we have financial problems. In the meantime, there is little to cushion you against the costs. | Patient | 19 | 248 | 68.1 | 28 | 160 | 69.4 | -1.3 | 0.794 |
| Exercise and physical fitness | Since the transplant, exercise is very important to me. I enjoy it immensely. | Patient | 20 | 267 | 67.8 | **9** | 160 | **76.9** | -12.1 | **0.045** |
| Person-centeredness | Everything is concentrated on the person who is ill. Family members are simply expected to be able to cope with it and continue to function. There is nobody for them to turn to. Nobody is interested in how they are managing at home. | Micro | 21 | 260 | 66.2 | 17 | 160 | 75.6 | -9.4 | **0.040** |
| Trustful relationships | The general practitioner cannot make decisions for the specialist. | Micro | 22 | 276 | 65.9 | 32 | 158 | 53.2 | 12.7 | **0.009** |
| Financial pressure | It is an issue which concerns me. I rely on very expensive medicines for my entire life, and I ask myself "how can I finance this?" | Patient | 23 | 265 | 64.5 | 20 | 159 | 74.8 | -10.3 | **0.027** |
| Continuity of care | Prior to the transplant there is too little information about what happens afterwards. | Meso | 24 | 266 | 63.5 | **8** | 159 | **81.1** | -17.6 | **<0.001** |
| Financial pressure | I would like to use my work to finance my life, but it is barely possible. | Patient | 25 | 256 | 62.1 | 26 | 160 | 71.3 | -9.2 | 0.056 |
| Organization of care | The timing of the consultation is so tight that you barely have time to ask your own questions. | Meso | 26 | 276 | 59.4 | 12 | 155 | 78.7 | -19.3 | **<0.001** |
| Trustful relationships | In hospital they said I should go to my GP. But he is so overwhelmed with my case that it makes me even more uncertain, and I have lost confidence in the hospital and in my GP. | Micro | 27 | 251 | 57.0 | **7** | 160 | **82.5** | -25.5 | **<0.001** |
| Trustful relationships | What I say is not taken seriously by the doctors. It is brushed aside or seems unimportant. | Micro | 28 | 260 | 56.9 | 23 | 160 | 73.1 | -16.2 | **0.001** |
| Peer contact | It did me a lot of good being with like-minded people. We had the same problems and could talk about the same things. | Micro | 29 | 269 | 56.9 | 22 | 160 | 73.1 | -16.2 | **<0.001** |
| Medication taking | I don’t understand why the body can’t accept a new organ without anti-rejection drugs […] I’d be really happy if I didn’t have to take anti-rejection drugs, because they can cause other problems in the long term. | Patient | 30 | 253 | 56.1 | 33 | 156 | 49.4 | 6.7 | 0.183 |
| Work and finances | As a self-employed person, if I don't work there is no money coming in. And I haven't received any social benefits either. | Patient | 31 | 221 | 56.1 | 25 | 160 | 71.3 | -15.2 | **0.003** |
| Medication taking | The drugs cause side effects that are very embarrassing like hair loss and high hairiness, it’s hard to live with it. | Patient | 32 | 262 | 55.0 | 27 | 158 | 69.6 | -14.6 | **0.003** |
| Medication taking | I’d like there to be more alternative medicine […] I think alternative medicine would be a really important addition to this field. | Patient | 33 | 263 | 53.6 | 34 | 156 | 36.5 | 17.1 | **0.001** |
| Pregnancy | Having a child after my transplant is important to me. I am wondering how the research in this domain is progressing. | Patient | 34 | 226 | 46.9 | 31 | 159 | 64.8 | -17.9 | **0.001** |
